# Complications of influenza in 272 adult and pediatric patients in a German university hospital during the seasonal epidemic 2017–2018

**DOI:** 10.1007/s10354-021-00884-0

**Published:** 2021-09-28

**Authors:** Hilte F. Geerdes-Fenge, Saskia Klein, Hans-Martin Schuldt, Micha Löbermann, Kerstin Köller, Jan Däbritz, Emil Christian Reisinger

**Affiliations:** 1 Department of Tropical Medicine and Infectious Diseases, Centre of Internal Medicine, Rostock University Medical Centre, Ernst-Heydemann-Str. 6, 18057 Rostock, Germany; 2 Institute of Medical Microbiology, Virology and Hygiene, University Medical Centre, Rostock, Schillingallee 70, 18057 Rostock, Germany; 3 Department of Paediatrics, Rostock University Medical Centre, Ernst-Heydemann-Str. 8, 18057 Rostock, Germany

**Keywords:** Cardiac insufficiency, Vaccination, Pneumonia, Myocardial infarction, Perimyocarditis

## Abstract

**Background:**

The influenza season 2017–2018 of the northern hemisphere was the highest since 2001 and was caused predominantly by influenza B virus.

**Methods:**

We performed a retrospective analysis of all patients in a university hospital in northern Germany with laboratory-confirmed influenza during the winter season 2017–2018 and analyzed underlying conditions, complications, and outcome.

**Results:**

A total of 272 cases of influenza were diagnosed: 70 influenza A (25.7%), 201 influenza B (73.9%), and 1 co-infection. Of 182 adults, 145 were hospitalized, 73 developed pneumonia, 11 developed myocardial infarction, two a transient ischemic attack, one a stroke, and one perimyocarditis. Eleven of the 145 hospitalized adult patients (7.6%) died, ten of them because of pneumonia. All of them had preexisting diseases. Pneumonia was associated with a mortality of 13.7%. Underlying cardiac insufficiency was correlated with higher mortality (7/51 with versus 4/126 patients without cardiac insufficiency; *p* < 0.05). Ninety cases of influenza were diagnosed in 89 children (30 A, 60 B), one child had first influenza B, then influenza A. Twenty-eight children (31%) were hospitalized, 15 children developed one or more complications (lower respiratory tract infections, meningeal irritations, febrile seizures, otitis media, myositis). No child died. Influenza vaccination status was known in 149 adult patients, pneumonia occurred more frequently in non-vaccinated individuals (43/90; 47.8%) than in vaccinated patients (18/59; 30.5%, *p* < 0.05).

**Conclusion:**

Patients with influenza should be monitored for secondary pneumonia and myocardial infarction, and vaccination should be enforced especially in patients with coronary heart disease and cardiac insufficiency.

## Introduction

Seasonal influenza, caused by influenza A or B viruses, is estimated to cause 300,000 to 650,000 deaths globally every year [1]. In 2018, an estimated 109.5 million cases of seasonal influenza occurred in children younger than 5 years worldwide, with an estimated death rate of 1:3150, nearly exclusively in developing countries [2]. In Germany, data on seasonal influenza are obtained by the Robert Koch Institute (RKI). During the influenza season 2017–2018, a total of 334,000 cases of laboratory-diagnosed influenza were reported to the RKI by local health authorities, more than in any prior season since 2001 when reporting began according to the German Infection Protection Act (IfSG). Influenza virus B was responsible for the majority of cases (68%). A total of 1674 deaths due to influenza were reported, more than in any other year since 2001 [3]. The aim of this study was to address whether the predominant influenza B strain caused more complications than influenza A in adults and in children, and whether prior vaccination reduced the rate of complications or death.

## Patients and methods

### Study design and patient identification

We performed a retrospective analysis of all patients with laboratory-confirmed infection with influenza A or B viruses between December 2017 and May 2018 in the Rostock University Medical Center, a 1100-bed tertiary referral hospital. All hospitalized patients as well as patients admitted to the emergency room or treated in outpatient clinics were included. The ethical committee of the Rostock University Medical Center approved the study (A 2018-0039). Informed consent was not required due to the observational nature of the study. Diagnostic procedures of respiratory specimens were performed using antigen immunoassays (QuickVue Influenza A + B Test, Quidel Corporation, San Diego, USA) and molecular analysis (GeneXpert® Flu/RSV XC-System, Cepheid, Sunnyvale, USA), according to the manufacturers’ protocols. If the antigen test was negative, a polymerase chain reaction (PCR) was performed. All patients with positive influenza antigen or positive influenza PCR were included. In case of repeated positive influenza tests, only the first test was included.

### Data collection

Two of the authors extracted clinical data from the electronic health records and the manual charts including demographics; comorbidities; prior influenza vaccination; presenting symptoms; duration from onset of symptoms to diagnosis; laboratory, microbiological, and radiographic findings; intensive care unit (ICU) admission; mechanical ventilation; hemodialysis; antimicrobial therapy; complications; and length of stay. The primary outcome was the occurrence of complications and in-hospital mortality.

### Definitions

Nosocomial influenza infection was defined as beginning of symptoms while a patient was in the hospital for ≥72 h prior to a positive test for influenza A or B. Pneumonia was defined as occurrence of new infiltrates on chest X‑ray or computed tomography (CT) scan described by a radiologist. Myocardial infarction was defined as signs of cardiac ischemia and increase of troponin T above the upper level of normal range. ST-elevation myocardial infarction (STEMI) was diagnosed if clinical signs of myocardial infarction were present and new ST elevation was detected.

### Statistical analysis

Sample demographic characteristics and clinical measures were summarized by descriptive statistics (frequency and percent for categorical variables; mean and standard deviation). The *t*-test was used for normally distributed data and the Mann–Whitney U test for non-normally distributed data. The association between virus type and severity of disease, complications, and death was analyzed using Pearson’s chi-square test. All statistical analysis was performed with statistical package and service solutions (SPSS; IBM SPSS Statistics 25.0, Chicago, Illinois, USA) software. All analyses assumed a 5% level of significance.

## Results

### Baseline characteristics

During the winter season 2017–2018, a total of 934 influenza tests were performed in the Rostock University Medical Center, Germany, between October 2017 and May 2018. Of these, 293 were positive (31.4%), 82 for influenza A (28.0%) and 211 for influenza B (72.0%), corresponding to 272 cases, some of which were tested repeatedly. There were almost twice as many tests performed as in the previous season, 2016–2017, when 435 tests gave 91 positive results (20.9%), 87 for influenza A (95.6%) and four for influenza B (4.4%). While tests had been performed since October 2017, the first positive test was seen in January 2018: between January 5 and April 10, 2018, 272 patients were diagnosed with virologically confirmed influenza (Table 1). Twenty-three adult patients had a nosocomial influenza infection.

**Table 1:** Epidemiological data of 272 cases of influenza during the season 2017/2018

	All cases	Adults	Children
Number of all cases	272	182	90
Influenza A, *n* (%)	70 (25.7%)	40 (21.9%)	30 (33.3%)
Influenza B, *n* (%)	201 (73.9%)	141 (77.5%)	60 (66.6%)
Influenza A + B, *n* (%)	1 (0.37%)	1 (0.5%)	0
Male sex, *n* (%)	155 (57.0%)	104 (57.1%)	51 (56.6%)
Hospitalized patients	173 (63.6%)	145 (79.7%)	28 (31.1%)
Age (mean ± SD)	48.4 ± 32.1 years	68.6 ± 17.2 years	7.61 ± 4.66 years
Age range	0–94 years	18–94 years	12 days–16 years
Body weight (mean ± SD)	–	79.4 ± 19.7 kg	25.9 ± 16.4 kg
Body height (mean ± SD)	–	1.69 ± 0.10 m	1.18 ± 0.34 m
Length of hospital stay (mean ± SD)	–	10.36 ± 8.76 days	2.82 ± 2.07 days
Any complication, *n* (%)	139 (51.1%)	124 (68.1%)	15 (16.7%)
Mortality, *n* (%)	11 (4.0%)	11 (6.0%)	0

### Adult patients

A total of 182 adult patients were included in the study, 145 were hospitalized. Complications occurred in 124 adult patients (68%): 73 developed pneumonia, bacterial pathogens were established in six cases (three *S. pneumoniae*, two *S. aureus*, one co-infection with *E. coli* and *Pseudomonas aeruginosa*). Seventeen patients were diagnosed with sepsis (14 of them associated with pneumonia), bacterial pathogens were identified in six cases (two *S. aureus*, two enterococci, one *S. pneumoniae*, one *E. coli*). Eleven patients developed myocardial infarction, two of them STEMI and nine patients non-STEMI. The infarction occurred within 0–7 days after the beginning of the influenza symptoms, three had influenza A, eight had influenza B, none of them died. One 74-year-old man with influenza B developed perimyocarditis complicated by nonfatal cardiogenic shock. Other complications were transient ischemic attacks in two patients and ischemic stroke in one patient who also had a myocardial infarction.

Adult patients with influenza A were significantly younger (62.62 ± 18.47 years) than patients with influenza B (70.27 ± 16.59 years, *p* = 0.013) and they were significantly more likely to be transmitted to the ICU (7/40 versus 7/141, *p* = 0.006) and mechanically ventilated (6/40 versus 6/141, *p* = 0.011). There was no significant difference between patients with influenza A versus influenza B in the overall complication rate (24/40 vs. 77/141, respectively, *p* = 0.544) or mortality rate (2/40 versus 9/141, respectively, *p* = 0.747).

Nine men and two women died 3–27 days after the onset of influenza symptoms, two of them with influenza A (5.0% of all influenza A patients), and nine with influenza B (6.4% of all influenza B patients). The cause of death was pneumonia in 10 patients and cardiac arrhythmia in one patient with acute exacerbation of chronic obstructive pulmonary disease (COPD) and gastrointestinal hemorrhage. All 11 patients who died had at least one underlying disease (see Table 2). Preexistent cardiac insufficiency was a significant risk factor for mortality, 7 of 51 patients with cardiac insufficiency (13.7%) died compared to 4 of 126 patients without cardiac insufficiency (3.2%, *p* = 0.008). Other significant risk factors for mortality were development of pneumonia, sepsis, acute renal failure, or intensive care treatment (see Table 2). Oseltamivir treatment was given to 94 of 182 (51.6%) adult patients.

**Table 2:** Comparison between survivors and non-survivors of 182 adult patients with influenza

	All adult patients (*n* = 182)	Survivors (*n* = 171)	Non-survivors (*n* = 11)	Significance
*Mean age* *±* *SD* *(age range in years)*	68.6 ± 17.2 (18–94)	68.05 ± 17.4 (18–93)	77.7 ± 12.4 (53–94)	*p* = 0.077
*Male patients*	104 (57.1%)	95 (55.6%)	9 (81.8%)	*p* = 0.094
*Influenza A*	40 (21.9%)	38 (20.9%)	2 (18.2%)	*p* = 0.747
*Influenza B*	141 (78.0%)	132 (72.5%)	9 (81.8%)	
*Influenza A* *+* *B*	1 (0.5%)	1 (0.6%)	0	–
*Medical history*				
Cardiac insufficiency	51 (28%)	44 (25.7%)	7 (63.6%)	*p* = 0.008*
Arterial hypertension	125 (68.7%)	118 (69.0%)	7 (63.6%)	*p* = 0.599
COPD or asthma	50 (27.5%)	46 (26.9%)	4 (36.4%)	*p* = 0.631
Chronic kidney disease	63 (34.6%)	59 (34.5%)	4 (36.4%)	*p* = 0.956
Coronary heart disease	59 (32.4%)	56 (32.7%)	3 (27.3%)	*p* = 0.660
Immunosuppression	28 (15.4%)	25 (14.6%)	3 (27.3%)	*p* = 0.282
Previous stroke	25 (13.7%)	23 (13.4%)	2 (18.2%)	*p* = 0.690
Diabetes mellitus	58 (31.9%)	56 (32.7%)	2 (18.2%)	*p* = 0.287
Malignant disease	17 (9.3%)	15 (8.8%)	2 (18.2%)	*p* = 0.319
Chronic liver disease	16 (8.8%)	15 (8.8%)	1 (9.1%)	*p* = 0.995
*Complications*				
Myocardial infarction	11 (6.04%)	11 (6.4%)	0	*p* = 0.378
Pneumonia	73 (40.1%)	63 (36.8%)	10 (90.9%)	*p* = 0.001*
Sepsis	17 (9.3%)	9 (5.3%)	8 (72.7%)	*p* = 0.001*
Acute renal failure	46 (25.3%)	39 (22.8%)	7 (63.6%)	*p* = 0.003*
Treatment in ICU	14 (7.7%)	10 (5.8%)	4 (36.4%)	*p* = 0.001*
*Influenza vaccination*	59 (32.4%)	55 (32.2%)	4 (36.4%)	*p* = 0.820

Values given as* n* (%) unless stated otherwise

*COPD *chronic obstructive pulmonary disease, *ICU *intensive care unit

*Statistically significant *p*-value

### Vaccination status in adult patients

Information on influenza vaccination status was available for 149 adults (Table 3), 55 received a trivalent and four a tetravalent vaccine. Pneumonia occurred significantly less frequently in vaccinated persons than in non-vaccinated persons (18/59 = 30.5% versus 47/90 = 52.2%, *p* = 0.036). The overall complication and mortality rate of unvaccinated patients was higher than in vaccinated adults; however, these differences failed to reach significance. The rate of vaccination was significantly higher in patients ≥60 years (54/115; 47.0%) than in patients <60 years (5/34; 14.7%; *p* = 0.001). However, only 57/139 (41.0%) of all patients with any indication for vaccination (age ≥60 years and/or medical indication for vaccination) had received a prior seasonal influenza vaccine.

**Table 3:** Data on prior influenza vaccination in 149 adult patients with influenza

	Vaccinated	Non-vaccinated	Significance
Number of patients (*n* = 149)	59	90	–
Influenza A (*n* = 34)	9 (15.3%)	25 (27.8%)	*p* = 0.137
Influenza B (*n* = 114)	50 (84.7%)	64 (71.1%)	
Influenza A + B (*n* = 1)	0	1 (1.1%)	
Hospitalization (*n* = 124)	50 (84.7%)	74 (82.2%)	*p* = 0.687
Overall complications (*n* = 107)	40 (67.8%)	67 (74.4%)	*p* = 0.378
Pneumonia (*n* = 61)	18 (30.5%)	43 (47.8%)	*p* = 0.036*
Mortality rate (*n* = 11)	4 (6.8%)	7 (7.8%)	*p* = 0.820

*Statistically significant *p*-value

Whereas 9/59 (15.2%) of the vaccinated patients were infected with influenza A virus, the others (50/59, 84.7%) had influenza B. Of those vaccinated with a tetravalent vaccine, one had influenza A und three influenza B viruses.

### Pediatric patients

Ninety influenza cases were diagnosed in 89 children (see Table 1). One 7‑year-old boy developed influenza B, followed by influenza A 4 weeks later; he had no complications. Twenty-eight children (31.1%) were hospitalized (11 with influenza A, 17 with influenza B). Fifteen children (median age 5 years, range 0–16) developed one or more complications: five children had lower respiratory tract infections (one 11-month-old boy with respiratory failure due to influenza A and pneumonia with *S. pneumonia*, three children aged 3, 4, and 5 years with bronchitis due to influenza A, and one 10-year-old girl with influenza B pneumonia). Six children had a meningeal irritation (2, 2, 4, 14, 15, and 16 years), three had febrile seizures (2, 6, and 10 years), one an otitis media (9 years), and one a myositis (6 years). Complications were more frequently observed with influenza A (9/11; 81.8%, versus influenza B: 6/17; 35.3%, *p* < 0.05). Five patients had to be treated in the ICU: a 12-day-old boy with fever (influenza B), a 5-year-old immunosuppressed girl with high fever of 41 °C (influenza B), an 8-year-old boy with severe epistaxis (influenza B), a 10-year-old girl with febrile seizures and respiratory failure due to influenza B pneumonia treated with high-flow oxygen therapy, and one 16-year-old girl with meningeal irritation (influenza A). No child was mechanically ventilated. One child was treated with oseltamivir. No child died due to influenza. Children with influenza A were significantly younger than those with influenza B (6.13 ± 5.16 years versus 8.35 ± 4.24 years, *p* = 0.014).

## Discussion

In the winter season 2017–2018, 73.9% of all influenza cases in a large University medical center located in North Germany were caused by influenza virus B. This corresponded to national data of the seasonal influenza epidemic in Germany (week 52/2017 to week 14/2018), with predominance of influenza B in 68%, while influenza A(H1N1)pdm09 was present in 28% and influenza A(H3N2) in 4%. Of all influenza B-viruses, 99% belonged to the Yamagata lineage that was not included in the trivalent vaccine [3]. The same distribution of Influenza subtypes can be assumed in the analyzed patients. The predominance of influenza B, especially of the Yamagata lineage, was seen worldwide during summer 2017 in the southern hemisphere and during the 2017–2018 winter season in the northern hemisphere, especially in Europe and Northern Asia, with a higher activity of influenza-like illness than in previous years [4].

The effect of prior vaccination on outcome in our study was not as notable as expected. Prior vaccination corresponded with a lower rate of influenza A (15.3% in vaccinated versus 27.8% in non-vaccinated patients). Vaccinated patients with influenza had a significantly lower rate of pneumonia compared to non-vaccinated patients; however, the difference in mortality rate, myocardial infarction, or total complication rate was not significant. This may be due to the facts that the vaccine did not cover the influenza B Yamagata lineage, and that predominantly patients with risk factors who have a higher mortality risk received influenza vaccination, while only few patients without underlying diseases had received vaccination. Overall, the vaccination rate was low: only 41% of all analyzed patients with any indication for vaccination (age ≥60 years and/or medical reasons for vaccination) had received a prior seasonal influenza vaccine.

In our study, adult patients with influenza A were more likely to be treated in the ICU and ventilated; however, there were no significant differences between influenza A or B with regard to overall complication rate or mortality in adults. Children with influenza A were significantly younger and had significantly more complications than children with influenza B. In accordance with our findings, a study from Finland in 358 children ≤13 years [5] noted that children with influenza A(H3N2) were significantly younger than those with A(H1N1) or B. However, they found no differences in clinical presentation, duration of illness, and frequency of complications between children with A/H1N1, A(H3N2), and B infections. Similarly, a recent study analyzing influenza-related pediatric deaths in the USA between 2010 and 2018 found no difference between infection with influenza A or B [6]. Complications of influenza in children in our study, like lower respiratory tract infection, meningeal irritation, febrile seizures, otitis media, and myositis, were comparable to data from other studies [7, 8]; in our study, no child died.

The main complication that still drives influenza mortality since the 1918 influenza pandemic is pneumonia [9], either viral or secondary. In our study, pneumonia was the most important complication in 40% of all adult patients with influenza. The mortality rate of pneumonia was 13.7% compared to the mortality rate of 0.9% in patients without pneumonia. The overall mortality rate was 6.0% in all 182 adult patients, the in-hospital mortality was 7.6% of 145 hospitalized adults. This rate corresponds to the in-hospital mortality rate of 6.7% out of 1539 influenza patients hospitalized in eight hospitals in central Germany during the influenza season 2017–2018 [10] and the in-hospital mortality rate of 8.2% out of 195 hospitalized patients in an Austrian tertiary care hospital [11]. In a University Hospital in Hamburg, the in-hospital mortality was 12.8%; this was due to a high percentage of referred patients from other hospitals for extracorporeal membrane oxygenation (ECMO) treatment [12].

Bacterial superinfection seems to be the most important pathogenic cause of mortality, most frequently caused by pneumococci or *S. aureus*, but also Gram-negative pathogens such as *Haemophilus influenzae* and *Pseudomonas aeruginosa* [13].

Our data underline the importance of cardiac insufficiency as a risk factor for mortality. Underlying cardiac insufficiency in our study was associated with a 4.3-times higher mortality rate than in patients without cardiac insufficiency. A large study found that influenza vaccination was associated with a 20% reduced risk for all-cause mortality in patients with heart failure and reduced ejection fraction [14]. Especially patients with type 2 diabetes [15] and elderly patients [16] benefit from a seasonal influenza vaccination, with a reduced hospitalization rate for cardiovascular events and stroke.

While the evidence of published case reports suggests that the most important extra-pulmonary complications of influenza are viral myocarditis and viral encephalitis [17], our data show that the major extra-pulmonary complication was myocardial infarction in 6% of all adult patients, while perimyocarditis occurred only in one patient. A study by Kwong et al. [18] indicated a significant association between influenza and myocardial infarction: the hospitalization rate for myocardial infarction was 6.05-times higher during the week after virologically confirmed influenza compared to the year before influenza and the remaining year after influenza, the odds ratio (OR) for myocardial infarction being 10.11 with influenza B and 5.17 with influenza A, while the OR was 3.51 with respiratory syncytial virus infections and 2.77 with other respiratory virus infections. Influenza vaccination reduced the risk of cardiovascular death in patients with coronary heart disease by about 50%, and cardiovascular events by about 43% [19].

While a large meta-analysis indicated that treatment with oseltamivir reduced the time to alleviation of all symptoms significantly and reduced lower respiratory tract complications [20], our data showed no significant positive effect of treatment with oseltamivir with regard to complications, death, or length of hospitalization. However, there may have been a bias, since oseltamivir is given preferably to more severely ill patients with a higher risk of complications.

The strength of our study was the inclusion of all adult and pediatric patients with influenza in a large university hospital and the assessment of all complications. The limitations of the study were the retrospective study design and the absence of a defined testing algorithm for influenza. Although the general awareness in the emergency room and in the hospital was high, patients with no or only mild symptoms might not have been tested for influenza and therefore have been missed in the study.

## Conclusion

Influenza continues to cause high morbidity and mortality. Pneumonia is the most relevant risk factor for influenza mortality. The major extra-pulmonary complication is myocardial infarction. Vaccination rates for influenza should be increased in all age groups, especially in patients with preexisting diseases such as cardiac insufficiency and coronary heart diseases.
